# Glucose, Insulin and Oxygen Modulate Expression of Serotonin-Regulating Genes in Human First-Trimester Trophoblast Cell Line ACH-3P

**DOI:** 10.3390/biomedicines11061619

**Published:** 2023-06-02

**Authors:** Maja Perić, Marina Horvatiček, Veronika Tandl, Ivona Bečeheli, Alejandro Majali-Martinez, Gernot Desoye, Jasminka Štefulj

**Affiliations:** 1 Division of Molecular Biology, Ruđer Bošković Institute, HR-10000 Zagreb, Croatia; maja.peric@irb.hr (M.P.); marina.horvaticek@irb.hr (M.H.); ivona.beceheli@irb.hr (I.B.); 2 Department of Obstetrics and Gynecology, Medical University of Graz, A-8036 Graz, Austria; veronika.tandl@medunigraz.at (V.T.); alejandro.majali@universidadeuropea.es (A.M.-M.); 3 Departamento de Medicina, Facultad de Ciencias Biomédicas y de la Salud, Universidad Europea de Madrid, 28670 Madrid, Spain

**Keywords:** gestational diabetes mellitus, obesity, 5-HT, 5-HTT, placenta, human trophoblasts, early pregnancy, hyperglycemia, hyperinsulinemia, hypoxia

## Abstract

Serotonin signaling plays an important role in regulating development and functions of the placenta. We hypothesized that metabolic disturbances associated with maternal obesity and/or gestational diabetes mellitus (GDM) affect placental serotonin homeostasis. Therefore, we examined the effects of high glucose (25 mM) and insulin (10 nM)—two hallmarks of maternal obesity and GDM—on mRNA expression of key regulators of serotonin homeostasis, including serotonin transporter (*SERT*), tryptophan hydroxylase 1 (*TPH1*), and monoamine oxidase A (*MAOA*), in the first-trimester trophoblast cell line ACH-3P, focusing on oxygen levels characteristic of early human placental development. Glucose downregulated expression of *SERT* and *MAOA* independently of oxygen level and upregulated expression of *TPH1* at 6.5% oxygen but not at 2.5% oxygen. Compared to 6.5% oxygen, 2.5% oxygen upregulated *SERT* and downregulated *TPH1* expression, with no effect on *MAOA* expression. Insulin upregulated *SERT* only at 2.5% oxygen but had no effect on *TPH1* and *MAOA* expression. These results suggest that maternal metabolic alterations in early pregnancy may be a driving force for changes in placental serotonin homeostasis.

## 1. Introduction

Serotonin (5-hydroxytryptamine, 5-HT) is a biogenic monoamine that plays a key role in regulating a wide range of physiological functions [1]. Disturbed serotonin homeostasis has been implicated in the pathogenesis of many complex diseases, including metabolic disorders such as obesity [2]. During pregnancy, serotonin plays a role in regulating maternal pancreatic beta-cell mass [3] and insulin secretion [4]. These physiological mechanisms, if dysfunctional, can contribute to the development of gestational diabetes mellitus (GDM).

Maternal obesity and GDM are characterized by a spectrum of metabolic, endocrine, inflammatory, and oxidative stress abnormalities, as evidenced by changes in the levels of the respective markers in blood plasma and extracellular fluids of different organs, including the placenta [5]. GDM, which is defined as glucose tolerance impairment first recognized during pregnancy [6], is primarily characterized by maternal hyperglycemia. In fact, GDM is the most common cause of hyperglycemia during pregnancy. Maternal obesity, on the other hand, is often associated with hyperinsulinemia [7]. Both hyperglycemia and hyperinsulinemia have been independently linked to impaired development of the placenta during early pregnancy, such as reduced trophoblast proliferation [8] or increased placental growth [9]. Furthermore, both GDM [10] and obesity [11] have been associated with placental hypoxia, which can negatively impact the development and function of the placenta, further contributing to adverse pregnancy outcomes [8,10].

The placenta is a fetal organ that begins to develop within the first few days after conception and continues to grow rapidly until the end of the first trimester/beginning of the second trimester of the pregnancy [12]. During this period, the placenta is also exposed to a progressive increase in oxygen tension from severely hypoxic (2–3% O_2_) to physiologically normoxic (about 6–10% O_2_) levels [13]. This change, which occurs due to the establishment of the blood flow in the intervillous space, is associated with increased shear and oxidative stress and induces cellular signals crucial for the placental function [14,15]. During this critical period of development, the placenta is most vulnerable to changes and disturbances in its surroundings [16]. Trophoblasts, which include villous cytotrophoblasts and the syncytiotrophoblast, are in direct contact with maternal blood and are therefore most susceptible to maternal influences. The placenta is capable of adapting to changes in the maternal environment, particularly in response to altered maternal metabolism, in order to protect the developing embryo/fetus from potential harm [17]. However, if the adaptive capacity of the placenta is overwhelmed, the altered maternal environment can lead to adverse outcomes for fetal growth and development, as well as for the long-term health of the child.

In addition to various other physiological processes, serotonin contributes to regulating placental development and function [18]. Specifically, studies in experimental models of the human placenta have shown that the placental serotonin system is involved in molecular pathways that regulate cellular processes such as trophoblast proliferation, differentiation, and survival [19], as well as placental-umbilical blood flow [20] and endocrine functions of the placenta [21]. In addition, studies in animal models have implicated the placental serotonin system in the regulation of embryonic/fetal development, particularly neurodevelopment [22,23], as well as in nutrient transport across the placenta [24]. Accordingly, altered homeostasis of the placental serotonin system has been associated with placental structure abnormalities and neurobehavioral changes in the offspring [25].

The human placenta expresses several classes of proteins involved in the regulation of serotonin signaling, including transmembrane carriers, metabolic enzymes, and receptors for serotonin [18]. Of these, serotonin transporter (SERT; high-affinity serotonin transmembrane carrier), tryptophan hydroxylase 1 (TPH1; synthetic enzyme for serotonin), and monoamine oxidase A (MAOA; catabolic enzyme for serotonin) are key contributors for maintaining optimal serotonin levels and regulating placental serotonin activity.

Despite the demonstrated role of the placental serotonin system in mechanisms regulating developmental processes and placental functions, very little is known about the potential impact of GDM and maternal obesity on placental serotonin homeostasis. Therefore, the aim of this study was to investigate possible effects of the main components of the GDM/obesity-associated environment, i.e., increased glucose and insulin concentrations, on the expression of genes encoding key regulators of serotonin levels, namely SERT, TPH1 and MAOA. For that purpose, we used an established cell model of human first-trimester trophoblasts (ACH-3P) [26] and performed the experiments under the oxygen levels characteristic of the early placental environment (2.5% and 6.5% O_2_).

## 2. Materials and Methods

### 2.1. Cell Culture

All experiments were performed with the human first-trimester trophoblast cell line ACH-3P, established by fusion of human primary first-trimester trophoblasts (12th week of gestation) and the human choriocarcinoma cell line AC1-1. They have been thoroughly characterized in our laboratory at the Department of Obstetrics and Gynecology, Medical University of Graz, Graz, Austria, as previously described [26]. Cells were cultured in 75 cm^2^ flasks in Dulbecco’s modified Eagle’s medium (DMEM) containing low D-glucose and sodium pyruvate, or Ham’s F-12 medium, both containing L-glutamine (all from Thermo Fisher Scientific Inc., Foster City, CA, USA) and supplemented with 10% fetal bovine serum (FBS, GE Healthcare Life Science, Chicago, IL, USA) and 1% penicillin/streptomycin (Thermo Fisher Scientific Inc., Foster City, CA, USA). The cells were kept at 37 °C, in a humidified atmosphere containing 5% CO_2_ and 21% O_2_ in a standard cell culture incubator, or 5% CO_2_ and 2.5% or 6.5% O_2_ in the XVIVO incubation system (BioSpherix, Ltd., Parish, NY, USA).

### 2.2. Glucose and Insulin Treatments

For glucose and insulin treatment experiments, ACH-3P cells were seeded on 6-well plates and cultured under low-serum conditions (2% FBS). This approach aimed to provide more controlled and standardized experimental conditions by mitigating potential interference from insulin, glucose, and serotonin present in serum. The time points for treatments were selected based on published studies on the effects of glucose and insulin in trophoblast cellular models [27,28] and the results of our preliminary experiments.

For the glucose treatment experiments, the ACH-3P cells were cultured for 4 days prior to treatment under low-serum conditions (2% FBS) in DMEM at 37 °C in a humidified atmosphere containing 5% CO_2_ and 2.5%, 6.5% or 21% O_2_. The cells were then incubated for 24 h or 72 h in DMEM containing D-glucose at a concentration of 5.5 mmol/L (control, normoglycemia) or 25 mmol/L (hyperglycemia). For osmotic control, 19.5 mmol/L L-glucose (Sigma-Aldrich, St. Louis, MO, USA) was added to DMEM containing 5.5 mmol/L D-glucose. Glucose concentrations used to mimic normoglycemia and hyperglycemia are common in studies on the role of diabetic glucose levels in different cellular models (e.g., [29]) and were also used in our previous studies with the ACH-3P cell line [8].

For the insulin treatment experiments, the ACH-3P cells were cultured for 2 days before treatment under low-serum conditions (2% FBS) in Ham’s F-12 medium, and subsequently incubated in the absence (control) or presence of 10 nM insulin (Calbiochem, EMD Chemicals, San Diego, CA, USA) at 2.5% and 6.5% O_2_ for 24 and 48 h. Insulin concentration was chosen based on our previous dose-response experiments on ACH-3P cells, showing that the respective concentration mimicked hyperinsulinemia in our experimental setup [27]. This concentration also corresponds to the physiologically relevant postprandial concentration of insulin [30].

### 2.3. RNA Extraction and Gene Expression Analysis

Total RNA from ACH-3P cells was extracted using the AllPrep DNA/RNA/miRNA Universal Kit (Qiagen, Hilden, Germany) according to the manufacturer’s protocol, including an optional on-column DNase treatment step. RNA was reverse-transcribed using the LunaScript RT SuperMix Kit (New England BioLabs, Ipswich, MA, USA) following the manufacturer’s instructions.

Real-time quantitative PCR (RT-qPCR) assays were performed on the CFX384 Touch Real-Time PCR Detection System (Bio-Rad Laboratories, Hercules, CA, USA) using the SsoAdvanced Universal SYBR Green Supermix (Bio-Rad Laboratories, Hercules, CA, USA), according to the manufacturer’s recommendations. qPCR reactions for the genes of interest were prepared with 40 ng of cDNA, while the reactions for the reference genes included 10 ng cDNA per reaction. Primer sequences (Table A1) were obtained from the literature [31,32,33,34,35] and purchased from Metabion (Planegg, Germany). RT-qPCR assays were run in duplicate or triplicate. Specificity of qPCR amplicons was verified by melting curve analysis. The stability of genes analyzed was evaluated using the RefFinder [36]. Among three potential reference genes tested (listed in Table A1), tyrosine 3-monooxygenase/tryptophan 5-monooxygenase activation protein zeta (YWHAZ) was the most stable one and was, therefore, used for normalization. Relative expression levels were calculated using the comparative Cq (ΔΔCq) method.

### 2.4. Analysis of SERT Methylation by Bisulfite Pyrosequencing

We additionally examined whether high glucose affects methylation of CpG sites in regulatory regions of *SERT*, as suggested by our previous clinical study [37]. DNA methylation was quantified at 9 CpG sites in the promoter and 14 CpG sites in intron 1 of *SERT* (Table A2) by bisulfite pyrosequencing. Cellular DNA was isolated from ACH-3P cells using AllPrep DNA/RNA/miRNA Universal Kit (Qiagen, Hilden, Germany) according to the manufacturer’s protocol. Bisulfite conversion was performed with 800 ng DNA per sample, using the EZ DNA Methylation-Gold Kit (Zymo Research, Irvine, CA, USA). Regions of interest were amplified using PyroMark PCR Kit (Qiagen, Hilden, Germany), and pyrosequencing was conducted on the PyroMark Q24 Advanced Pyrosequencing System with PyroMark Q24 Advanced CpG Reagents (both from Qiagen, Hilden, Germany), all following the manufactures’ recommendations. Primers used in the analysis are listed in Table A3. All assays included a negative control and a reference sample. Pyrosequencing quality control was performed using PyroMark Q24 Advanced Software (version 3.0.0, Qiagen, Hilden, Germany). The methylation levels of the CpG sites in each region were positively correlated, so the average methylation for the promoter and intron 1 region was used in the analyses.

### 2.5. Statistics

Statistical analyses were performed with GraphPad Prism 8 (GraphPad Software Inc., San Diego, CA, USA) and IBM SPSS Statistics 23.0 for Windows (SPSS Statistics, Chicago, IL, USA). Normal distribution of data was tested using the D’Agostino–Pearson normality test. Data were transformed using reciprocal square root (glucose experiment) or square root (insulin experiment) to achieve a normal distribution for statistical analysis and re-transformed for the presentation of the results. Outliers were screened for using the robust regression and outlier removal (ROUT) method with maximum false discovery rate (Q) set to 1% [37].

Analysis of covariance (ANCOVA) with passage number included as a covariate was performed to determine the effect of glucose/insulin treatment and oxygen level and their interactions. Sidak’s post hoc test was used to adjust for multiple comparisons. When there was a significant interaction, a subsequent ANCOVA was conducted for comparisons between groups; *p* < 0.05 was considered statistically significant.

## 3. Results

### 3.1. Effect of Glucose on the Expression of Serotonin-Regulating Genes

**Figure 1:**
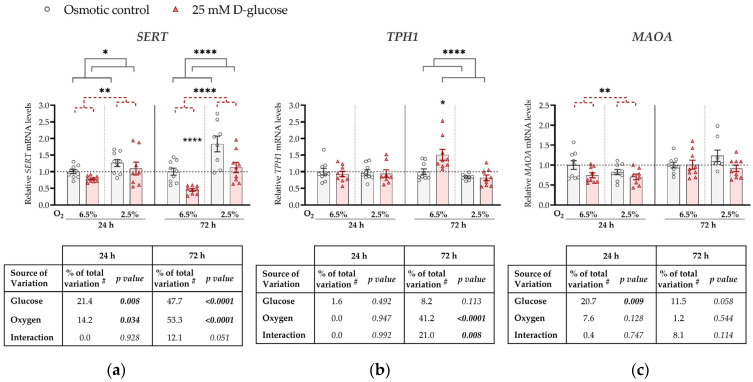
Effect of glucose and oxygen on mRNA expression of serotonin-regulating genes in ACH-3P cells. Cells were cultured in the presence of 5.5 mM D-glucose + 19.5 mM L-glucose (osmotic control, grey bars) or 25 mM D-glucose (hyperglycemia, red bars), at 6.5% O_2_ or 2.5% O_2_ for 24 h or 72 h. Relative mRNA expression levels of (**a**) *SERT*, (**b**) *TPH1* and (**c**) *MAOA* were determined by RT-qPCR and normalized to *YWHAZ* mRNA level. Results of three independent experiments, each run in triplicate, are shown. For each gene, data at each time point are presented relative to the mean of the osmotic control at 6.5% O_2_, which was arbitrarily set to 1.0; bars represent mean ± SEM. Statistical analysis used ANCOVA with cell passage as covariate and Sidak’s post hoc test to adjust for multiple comparisons. * *p* < 0.05, ** *p* < 0.01, **** *p* < 0.0001, red dashed line—main effect of glucose, gray line—main effect of oxygen. ^#^ proportion of total variance after controlling for the effect of the cell passage (partial η^2^).

**Figure 2:**
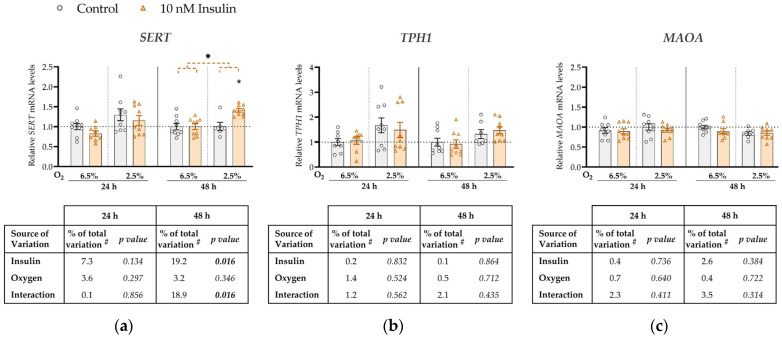
Effect of insulin and oxygen on mRNA expression of serotonin-regulating genes in ACH-3P cells. Cells were cultured in the absence (control, grey bars) or presence of 10 nM insulin (hyperinsulinemia, orange bars) at 6.5% O_2_ or 2.5% O_2_ for 24 h or 48 h. Relative mRNA expression levels of (**a**) *SERT*, (**b**) *TPH1* and (**c**) *MAOA* were determined by RT-qPCR and normalized to *YWHAZ* mRNA level. Results of three independent experiments, each done in duplicate or triplicate, are shown. For each gene, data are presented relative to the mean of the control sample at 6.5% O_2_, which was arbitrarily set to 1.0; bars represent mean ± SEM. Statistical analysis used ANCOVA with cell passage as covariate and Sidak’s test to adjust for multiple comparisons. * *p* < 0.05, orange dashed line—main effect of insulin. ^#^ proportion of total variance after controlling for the effect of the cell passage (partial η^2^).

**Figure A1:**
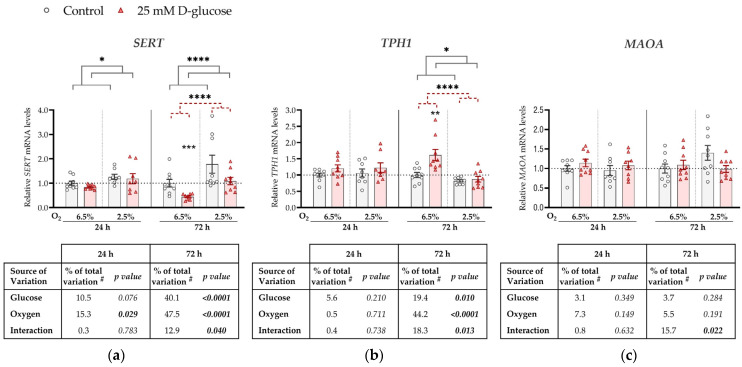
Effect of glucose and oxygen on mRNA expression of serotonin-regulating genes in ACH-3P cells. Cells were cultured in the absence (control, grey bars) or presence of 25 mM D-glucose (hyperglycemia, red bars) at 6.5% O_2_ or 2.5% O_2_ for 24 h or 72 h. Relative mRNA expression levels of (**a**) *SERT*, (**b**) *TPH1* and (**c**) *MAOA* were determined by RT-qPCR and normalized to *YWHAZ* mRNA level. Results of three independent experiments, each done in triplicate, are shown. For each gene, data at each time point are presented relative to the mean of the control samples at 6.5% O_2_, which was arbitrarily set to 1.0; bars represent mean ± SEM. Statistical analysis used ANCOVA with cell passage as covariate and Sidak’s post hoc test to adjust for multiple comparisons. * *p* < 0.05, ** *p* < 0.01, *** *p* < 0.001, **** *p* < 0.0001, red dashed line—main effect of glucose, gray line—main effect of oxygen. ^#^ proportion of total variance after controlling for the effect of the cell passage (partial η^2^).

Expression levels of *SERT*, *TPH1*, and *MAOA* mRNAs were compared between ACH-3P cells incubated (for 24 or 72 h) in the presence of 25 mM D-glucose (hyperglycemia) and (1) 5.5 mM D-glucose supplemented with 19.5 mM L-glucose (osmotic control; Figure 1) or (2) 5.5 mM D-glucose (control; Figure A1 in Appendix A). Both comparisons, using ANCOVA adjusted for cell passage, yielded similar results as described below.

Specifically, at both time points, *SERT* expression was significantly downregulated by the high glucose concentration (*p* = 0.008 and *p* < 0.0001, respectively) and upregulated by low oxygen level (2.5% O_2_) (*p* = 0.034 and *p* < 0.0001, respectively; Figure 1a). At 72 h, we observed a borderline significant (*p* = 0.051) interaction between glucose and oxygen on *SERT* expression. This trend might be driven by the more pronounced decrease in *SERT* expression by high glucose at 6.5% O_2_ (by 50%, *p* < 0.0001) than at 2.5% O_2_ (*p* = 0.186).

*TPH1* expression was not significantly affected at 24 h by either high glucose levels or low oxygen tension, nor did they significantly interact (Figure 1b). However, at 72 h, *TPH1* expression was downregulated by low oxygen tension (*p* < 0.0001). In addition, we observed a significant interaction between oxygen and glucose on *TPH1* expression at 72 h (*p* = 0.008). At this time point, high glucose increased *TPH1* expression at 6.5% O_2_ (by 17%, *p* = 0.019), while it had no effect at 2.5% O_2_.

*MAOA* expression was downregulated by high glucose concentrations, independently of oxygen, at 24 h (by 20%, *p* = 0.009; Figure 1c), while the effect of glucose was not significant at 72 h. While we observed an effect of oxygen on both *SERT* and *TPH1* expression, no effect of oxygen on *MAOA* expression was detected at either 24 h or 72 h.

### 3.2. Effect of Insulin on the Expression of Serotonin-Regulating Genes

To investigate the effect of insulin on mRNA expression of *SERT*, *TPH1* and *MAOA*, ACH-3P cells were cultured in the absence (control) or presence of 10 nM insulin (hyperinsulinemia) in conditions of 6.5% O_2_ or 2.5% O_2_, for 24 or 48 h (Figure 2).

*SERT* expression was not significantly affected by either insulin, oxygen or their interaction at 24 h (Figure 2a). However, at 48 h we observed a significant main effect of insulin on *SERT* mRNA levels (increase by 19%, *p* = 0.016). Furthermore, at 48 h there was a significant interaction between insulin and oxygen (*p* = 0.016; Figure 2a) such that insulin concentration significantly increased *SERT* expression only at 2.5% O_2_ (by 8%, *p* = 0.011). Neither oxygen, insulin, or their interaction had any effect on *TPH1* (Figure 2b) or *MAOA* (Figure 2c) expression at either 24 h or 48 h.

## 4. Discussion

This is the first study on the effects of glucose, insulin and oxygen, and their interaction, on expression levels of the key serotonin-regulating genes *SERT*, *TPH1* and *MAOA* using an established cellular model of first-trimester trophoblasts with emphasis on oxygen levels that are characteristic of early human placental development.

Our findings show that high glucose downregulates *SERT* expression (Figure 1a), while low oxygen (Figure 1a) or high insulin (Figure 2a) upregulate its expression. The downregulation of *SERT* expression by high glucose was evident at both oxygen levels, as evidenced by the statistically significant main effect of oxygen, although at a later time point, it might be more pronounced at 6.5% O_2_, as suggested by borderline significant interaction (*p* = 0.051) and results of the post hoc test. On the other hand, insulin upregulated *SERT* expression only at 2.5% O_2_. This suggests that early in pregnancy the trophoblast is already sensitive to maternal metabolic changes.

A previous study investigated the effect of high glucose on the function of SERT in the Caco-2 cell line derived from human colon adenocarcinoma [38]. Consistent with our results in ACH-3P cells, short-term exposure to high glucose (30–40 mM) decreased SERT-mediated serotonin uptake in Caco-2 cells [38]. However, long-term exposure (21–24 weeks) of Caco-2 cells to high glucose (30 mM) increased SERT-mediated serotonin uptake in Caco-2 cells [38]. This result is consistent with our previous clinical study, in which we found a positive correlation between the expression of *SERT* in human term placental tissue and glucose concentration in maternal fasting plasma during the second trimester of pregnancy [39]. Taken together, these findings suggest that the effects of high glucose on *SERT* expression depends on the duration of exposure, with short-term exposure resulting in downregulation and long-term exposure resulting in upregulation.

Insulin has been reported to regulate SERT trafficking from the endoplasmic reticulum to the cell membrane, but to have no effect on *SERT* mRNA expression in primary human trophoblasts at the end of pregnancy [40]. The respective expression results, obtained after 24 h of treatment at atmospheric oxygen tension (21%), are in agreement with our results showing no effect of insulin on *SERT* expression at the same time point regardless of oxygen tension. However, our results differed when treatment duration was extended to 48 h, when insulin increased *SERT* expression at 2.5% oxygen but had no effect at 6.5% oxygen. Accordingly, both duration of insulin exposure and oxygen tension clearly play a role in determining the effect of insulin on *SERT* expression. An additional possible explanation for the discrepancy between our results and those on primary term trophoblasts [40] is that the effect of insulin on *SERT* expression may vary depending on the period of pregnancy. In line with our finding of increased *SERT* expression at the 2.5% compared to the 6.5% oxygen level, *SERT* expression in the BeWo placental cell line was increased at 3% compared to 8% oxygen [41]. Hypoxia also increased *SERT* expression in pulmonary vascular smooth muscle cells [42]. In contrast, *SERT* expression in term placental explants was not affected by oxygen levels [41]. Importantly, placental explants were cultured at controlled oxygen levels for 24 h [41], whereas in our glucose treatment experiment, culture at controlled oxygen levels lasted for 5 or 7 days (including the pre-treatment period), which may have contributed to the different results. Potential mechanisms underlying sensitivity of the human *SERT* gene to oxygen tension may be explained by its promoter harboring motifs for binding of oxygen-sensitive transcription factors, e.g., hypoxia-inducible factor AP-1 [42,43].

In contrast to *SERT*, *TPH1* expression was increased by glucose treatment under conditions of 6.5% O_2_ (Figure 1b). While there are no studies examining the effects of glucose on *TPH1* expression in placental cellular models, high glucose profoundly induced *Tph1* expression in rat pancreatic islets, both in vitro and in vivo [44]. That study implicated the role of TPH1 enzyme in glucose-induced potentiation of β-cell function [44].

A previous study found *TPH1* expression to be upregulated by hypoxia in human pulmonary endothelial cells [45]. This result is in line with the finding that the murine *Tph1* promoter contains hypoxia-responsive elements (HRE) and a binding site for hypoxia-inducible factor AP-1 [45]. However, in our experiments, *TPH1* expression was decreased at 2.5% oxygen compared to 6.5% oxygen (Figure 1b). This suggests that the low-oxygen placental environment in early human pregnancy regulates *TPH1* expression by a mechanism different from the hypoxia-inducible response, i.e., that an alternative mechanism dominates over the hypoxia-inducible response at relatively subtle oxygen changes from 6.5% to 2.5%. Considering that the expression of *SERT*, which encodes a protein accounting for serotonin uptake into cells, increases at 2.5% compared to 6.5% oxygen, a possible mechanism responsible for the downregulation of the serotonin-synthesizing enzyme *TPH1* under such conditions could involve feedback regulation by increased intracellular serotonin levels. This speculation remains to be tested.

Like *SERT*, *MAOA* expression in ACH-3P cells was also downregulated in the presence of high glucose (Figure 1c). Outside pregnancy, short-term exposure to high glucose upregulates *MAOA* expression in the vascular rings of mammary arteries [46]. In addition to different experimental models, these effects were observed at ambient, i.e., 21%, oxygen levels, and cannot be directly compared to our results obtained at low oxygen levels.

Our results showed that *MAOA* expression was not affected by oxygen (Figure 1c). Based on the observations made on term placental tissues from pregnancies complicated by pre-eclampsia, a condition characterized by severe placental hypoxia, oxygen may affect the activity of placental MAOA rather than its expression [47,48]. Specifically, clinical studies found reduced MAOA activity [47,48], but no change in *MAOA* mRNA expression [47], in term placental tissue from pregnancies complicated with pre-eclampsia compared to normotensive pregnancies.

We showed no effect of insulin on *MAOA* (Figure 2b) or *TPH1* (Figure 2c) expression. To date, there are no studies on potential effects of insulin on *MAOA* or *TPH1* expression in trophoblasts or other cell types.

**Figure 3:**
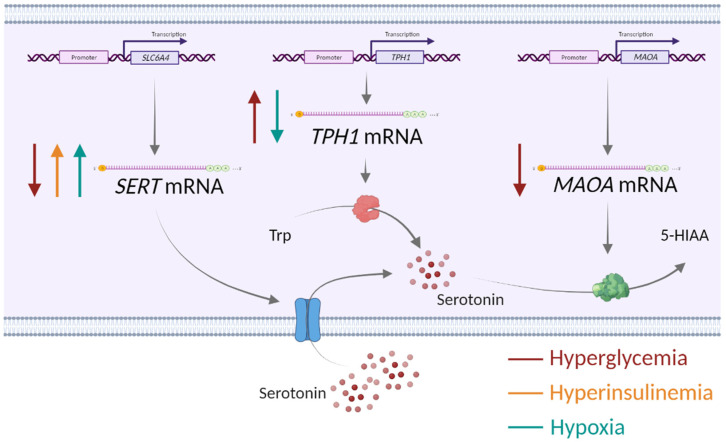
Impact of alterations in the placental environment associated with obesity and gestational diabetes mellitus—hyperglycemia, hyperinsulinemia and low oxygen—on the expression of serotonin-regulating genes in human first-trimester trophoblasts. SERT—serotonin transporter, TPH1—tryptophan hydroxylase 1, MAOA—monoamine oxidase A, Trp—tryptophan, 5-HIAA—5-hydroxyindolacetic acid, ↑ upregulation, ↓ downregulation. Created with Biorender.com.

Collectively, our findings on the opposing effects of glucose as well as of oxygen on *SERT* and *TPH1* expression, together with the concordant effects of glucose on *SERT* and *MAOA* expression (summarized in Figure 3), may suggest possible (patho)physiological implications. Specifically, as a speculation, glucose-induced downregulation of *SERT*, which mediates serotonin uptake into cells and is abundant at the syncytiotrophoblast surface facing maternal blood [49,50], may lead to increased levels of serotonin in the extracellular space and consequently to hyperactivation of serotonin receptors. Depending on receptor location, this may cause vasoactive effects on the maternal blood vessels or alter trophoblast proliferation, differentiation or migration processes [18]. On the other hand, *SERT* upregulation by hyperinsulinemia and hypoxia could lead to increased intracellular levels of serotonin with potential implications for biological effects via serotonylation of intracellular proteins [51,52]. The only effect observed on *MAOA* was its downregulation by glucose. MAOA activity is a major source of reactive oxygen species (ROS) in cells [53]. As shown previously in a rat bladder tumor cell line, reduced ROS production by MAOA inhibition reduced expression of proteins involved in glucose transport, such as GLUT1 [54]. Hence, one may speculate that *MAOA* downregulation in response to high glucose is an adaptive mechanism to reduce oxidative stress and/or glucose transport through the placenta.

**Figure 4:**
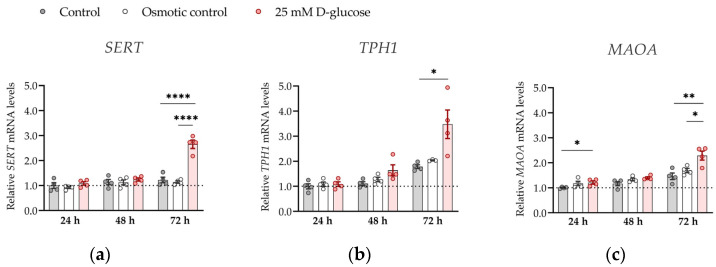
Effect of glucose on mRNA expression of serotonin-regulating genes in conditions of 21% O_2_. ACH-3P cells were cultured in the presence of 5.5 mM D-glucose (control, gray bars), 5.5 mM D-glucose + 19.5 mM L-glucose (osmotic control, white bars) or 25 mM D-glucose (hyperglycemia, red bars) for 24 h, 48 or 72 h at ambient oxygen. Relative expression levels of (**a**) *SERT*, (**b**) *TPH1* and (**c**) *MAOA* mRNA were determined by RT-qPCR and normalized to YWHAZ mRNA level. Results of two independent experiments, each done in duplicate, are shown. For each gene, data are presented relative to the mean of the control samples after 24 h, which was arbitrarily set to 1.00: bars represent mean ± SEM. Statistical analysis used ANCOVA with cell passage as covariate and Sidak’s post hoc test to adjust for multiple comparisons. * *p* < 0.05, ** *p* < 0.01 **** *p* < 0.0001.

To further strengthen the significance of our results, we investigated the effect of hyperglycemia on the expression of *SERT*, *TPH1* and *MAOA* genes at ambient (21%) oxygen levels (Figure 4). At ambient oxygen, high glucose had more profound effects on *SERT*, *TPH1,* and *MAOA* expression than at physiological oxygen levels, markedly increasing (by 1.5–2 times) expression of all three genes at 72 h (all *p* < 0.05). These results show that oxygen is clearly a major determinant of placental serotonin homeostasis to be considered in future in vitro studies.

**Figure 5:**
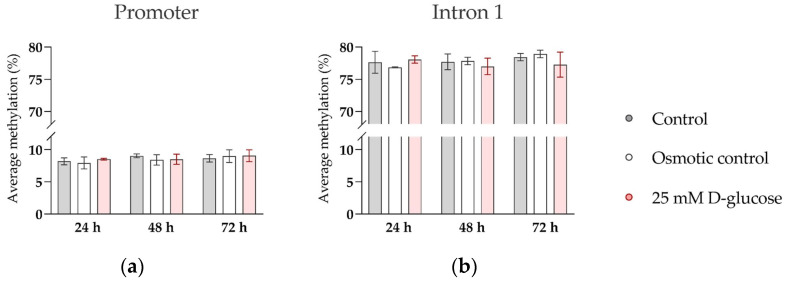
Effect of glucose on DNA methylation of *SERT* promoter and intron 1 region in ACH-3P cells. Cells were cultured in the presence of 5.5 mM D-glucose (control, gray bars), 5.5 mM D-glucose + 19.5 mM L-glucose (osmotic control, white bars), or 25 mM D-glucose (hyperglycemia, red bars) for 24 h, 48 h, or 72 h, at ambient oxygen level. Average DNA methylation across (**a**) 9 CpG sites in the promoter region and (**b**) 14 CpG sites in the intron 1 region, determined by bisulfite pyrosequencing, is shown (Mean ± SEM; *n* = 3–4).

While oxygen effects on *SERT* expression may be mediated by binding of oxygen-sensitive transcription factors, i.e., hypoxia-inducible factor AP-1, to the *SERT* promoter region [42,43], the molecular mechanisms by which glucose and insulin modulate expression of *SERT* are not known. In our previous clinical study on human term placental tissue, we found that maternal plasma glucose concentrations in the second trimester of pregnancy were negatively correlated with DNA methylation of the *SERT* promoter region, which in turn was negatively correlated with *SERT* expression [39]. This suggests that DNA methylation, an epigenetic mechanism sensitive to environmental cues [55], mediates the effect of long-term glucose exposure on *SERT* expression in the human placenta. To investigate whether the same mechanism also accounted for the effect of short-term glucose exposure on *SERT* expression in ACH-3P cells at the ambient oxygen level, where *SERT* expression was potently upregulated by glucose treatment for 72 h (Figure 4), we analyzed the methylation of the *SERT* promoter and intron 1 regions in representative cells. However, we did not detect any changes in DNA methylation between control and glucose-treated cells (Figure 5). This suggests that DNA methylation does not play a role in the effects of short-term glucose exposure on *SERT* expression in trophoblast cells.

Importantly, the proliferation of ACH-3P cells [56] as well as of several other trophoblast cell lines [57] was shown to be affected by hyperosmolarity. Our results show similar effects of hyperglycemia when compared to either osmotic control (Figure 1) or normoglycemic control (Figure A1), suggesting that the expression of *SERT*, *TPH1* and *MAOA* was directly affected by high glucose, and not elevated osmotic pressure. We have to point out that in our insulin treatment experiments, we have not observed a statistically significant effect of oxygen on the expression of any gene analyzed. This may be due to the different experimental set-up, mostly the shorter incubation time, i.e., 3 and 4 days in insulin treatment experiments compared to 5 and 7 days in glucose treatment experiments (including the pre-treatment periods).

Our study has several strengths. Firstly, while most studies of placental pathologies focus on term placentas, some of these pathologies originate due to placental dysfunction occurring in the early-pregnancy period. Thus, from a developmental standpoint, it is important to study early-pregnancy placentas using placental tissue or models. We chose to use first-trimester trophoblast-derived cell line ACH-3P, which is particularly suitable for in vitro experiments because it closely resembles primary human first-trimester trophoblasts, but compared to the primary trophoblasts, has proliferative properties [26]. It also expresses insulin receptors, essential for the present study [27].

Another advantage of our study is that we studied the effects of glucose and insulin in conditions of low oxygen tension, since early placental development takes place in a hypoxic environment [13], and *SERT* and *TPH1* expression were shown to respond differently in physiological, pathological or ambient oxygen levels [42,45]. Lastly, cellular properties in cell culture are dependent on the number of cell passages [58]. This is why including cell passage number as a covariate in statistical analyses adds strength to the observed effects.

A weakness of our study is that we analyzed only mRNA levels and did not examine protein levels or function. However, it needs to be noted that previous clinical studies in human placental tissue have reported consistent results for *SERT* mRNA and SERT protein levels in GDM [59] and for *MAOA* mRNA and MAOA protein levels in pre-eclampsia [47]. Furthermore, we used only one cell line, which may limit generalizability of the findings. Due to ACH-3P cells representing a male cell line, caution is needed when interpreting the current data, as the placental response to the altered environment varies between male and female fetuses [5] and the serotonin system generally exhibits sex-specific differences [18].

Therefore, future research should include protein levels/activity and additional genes and cell lines for more in-depth studies on functional consequences of the observed effects. This would contribute to a more comprehensive understanding of serotonin regulation in early human pregnancy but was beyond the scope of the present study.

It also remains to be elucidated whether the changes in expression of serotonin-related genes affected by the GDM/obesity-like environment are an adaptive response of the placenta to protect the embryo/fetus, or are the consequence of placental inability to adapt to the environmental changes. The latter could lead to some of the adverse outcomes for fetal growth and development associated with GDM/obesity. In the case of placental maladaptation, serotonin-regulating components in the placenta could become a potential target for prevention of the negative consequences of GDM and maternal obesity for the health of the child.

In conclusion, the results suggest that metabolic alterations linked to GDM and/or maternal obesity (hyperglycemia and hyperinsulinemia), together with oxygen changes, may be a driving force for changes in placental serotonin homeostasis occurring in the first trimester of pregnancy.

## Data Availability

The data presented in the study are available from the corresponding author upon reasonable request.

## Appendix B

**Table A1:** Sequences of gene-specific primers used in quantitative real-time PCR (RT-qPCR). f—forward, r—reverse.

Gene Symbol	Gene Name	Primer Sequence (5′–3′)	Amplicon (bp)	Source
*SERT*/ *SLC6A4*	serotonin transporter	f: AGATCCTGAGACGCATTGCT r: AACAGAGCGAAACTCCGAAA	504	[33]
*TPH1*	tryptophan hydroxylase 1	f: TGCAAAGGAGAAGATGAGAGAATTTAC r: CTGGTTATGCTCTTGGTGTCTTTC	114	[33]
*MAOA*	monoamine oxidase A	f: GAGCGGCTACATGGAAGGG r: TCACCTTCCCGAGACCATTTA	77	[31]
*YWHAZ*	tyrosine 3-monooxygenase/tryptophan 5-monooxygenase activation protein zeta	f: CCGTTACTTGGCTGAGGTTG r: AGTTAAGGGCCAGACCCAGT	143	[32]
*ACTB*	actin beta	f: TCCCTGGAGAAGAGCTACG r: GTAGTTTCGTGG ATGCCACA	131	[34]
*CK7*	cytokeratin 7	f: CCGTGCGCTCTGCCTATGGGG r: GCTCCAGAAACCGCACCTTGTCGAT	192	[35]

**Table A2:** Sequences of gene-specific primers used in *SERT (SLC6A4)* gene methylation analyses. f—forward, r—reverse, s—sequencing.

Region	Primer Sequence (5′–3′)	Amplicon (bp)	Source
promoter	f *: GGTTTTTAGGAGAGTAGGGAGTATATAGTT r: AAAAATCCTAACTTTCCTACTCTTTAACTT s: AAACTACACAAAAAAACAAAT	203	[60]
intron 1	f: GGGGAGGGGGATAGAAT r *: ACAACACTTTACTCAAAACCCTCTTTA s: GGGGAGGGGGATAGAAT	273	/
Intron 1	f: TTTTAAAGAGGGTTTTGAGTAAAGTG r *: TTTATATCAACCAAAACTCTCCCTTTACAT s: GGGTTTTGAGTAAAGTGT	150	/

* biotinylated primer.

**Table A3:** Coordinates of the analyzed CpG sites in *SERT (SLC6A4)* gene.

CpG Site	Location	GRCh38 Coordinates	NG_011747.2 Coordinates
1	promoter	30236157	4780
2	promoter	30236142	4795
3	promoter	30236126	4811
4	promoter	30236121	4816
5	promoter	30236102	4835
6	promoter	30236091	4846
7	promoter	30236089	4848
8	promoter	30236084	4853
9	promoter	30236072	4865
10	intron 1	30235532	5405
11	intron 1	30235527	5410
12	intron 1	30235519	5418
13	intron 1	30235512	5425
14	intron 1	30235504	5433
15	intron 1	30235490	5447
16	intron 1	30235482	5455
17	intron 1	30235475	5462
18	intron 1	30235472	5465
19	intron 1	30235457	5480
20	intron 1	30235272	5665
21	intron 1	30235247	5690
22	intron 1	30235203	5734
23	intron 1	30235201	5736
